# Supplementary figures and images for: Host immunity and the colon microbiota of mice infected with Citrobacter rodentium are beneficially modulated by lipid-soluble extract from late-cutting alfalfa in the early stages of infection
Source: PLoS One. 2020 Jul 16;15(7):e0236106. doi: 10.1371/journal.pone.0236106 (PMC7365448; doi:10.1371/journal.pone.0236106)

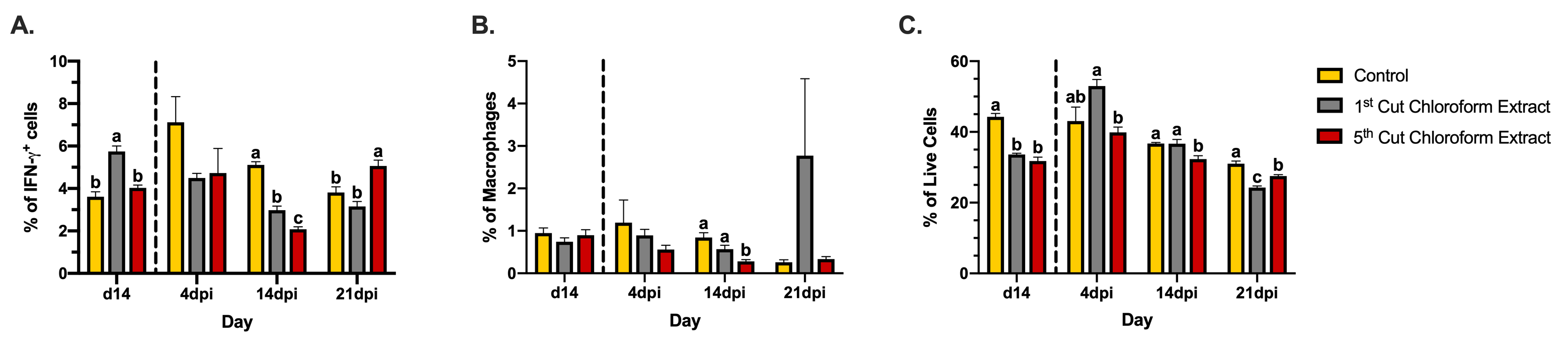

Supplement: S1 Fig — (A) IFN-γ+CD8+ cytotoxic T cells, (B) TNF-α+ macrophages, and (C) CD11b+ cells. Data are represented as the mean percentage of each cell type ± SEM. Bars with different superscripts are significantly different at P ≤ 0.05. The dashed line separates the end of the feeding-enrichment period and the start of the infection period. (TIFF) [file pone.0236106.s001.tiff]
