# Supplementary material for: Host immunity and the colon microbiota of mice infected with Citrobacter rodentium are beneficially modulated by lipid-soluble extract from late-cutting alfalfa in the early stages of infection
Source: PLoS One. 2020 Jul 16;15(7):e0236106. doi: 10.1371/journal.pone.0236106 (PMC7365448; doi:10.1371/journal.pone.0236106)
Supplement: S1 Table — (PDF) [file pone.0236106.s002.pdf]

S1 Table. Significantly different OTUs in the colon microbiota of healthy mice fed the control diet vs. 1st cutting chloroform extract at d14.

OTU	LDA effect size score	Treatment in which OTU is more abundant	p-value	Taxonomy
OTU 23	3.31	Control	0.037	*Lachnospiraceae A2*
OTU 42	2.87	1st cutting chloroform extract	0.010	*Lachnospiraceae_NK4A136_group*
OTU 44	2.55	Control	0.037	*Lachnospiraceae GCA-900066575*
OTU 46	2.37	1st cutting chloroform extract	0.010	*Lachnospiraceae uncultured*
OTU 56	3.08	1st cutting chloroform extract	0.004	*Lachnospiraceae_unclassified*
OTU 64	3.18	Control	0.016	*Lachnoclostridium*
OTU 74	3.55	Control	0.020	*Faecalibaculum*
OTU 88	3.17	Control	0.010	*Lachnospiraceae_NK4A136_group*
OTU 100	2.37	Control	0.037	*Lachnospiraceae_unclassified*
