# Supplementary material for: Host immunity and the colon microbiota of mice infected with Citrobacter rodentium are beneficially modulated by lipid-soluble extract from late-cutting alfalfa in the early stages of infection
Source: PLoS One. 2020 Jul 16;15(7):e0236106. doi: 10.1371/journal.pone.0236106 (PMC7365448; doi:10.1371/journal.pone.0236106)
Supplement: S2 Table — (PDF) [file pone.0236106.s003.pdf]

S2 Table. Significantly different OTUs in the colon microbiota of healthy mice fed the control diet vs. 5th cutting chloroform extract at d14.

OTU	LDA effect size score	Treatment in which OTU is more abundant	p-value	Taxonomy
OTU 1	4.67	5th cutting chloroform extract	0.025	*Muribaculaceae_ge*
OTU 10	4.53	Control	0.021	*Muribaculaceae_ge*
OTU 11	4.00	Control	0.037	*Lachnospiraceae_NK4A136_group*
OTU 14	3.28	5th cutting chloroform extract	0.037	*Muribaculaceae_ge*
OTU 23	3.06	Control	0.025	*Lachnospiraceae A2*
OTU 26	3.56	5th cutting chloroform extract	0.025	*Muribaculaceae_ge*
OTU 28	3.14	5th cutting chloroform extract	0.021	*Lachnospiraceae_unclassified*
OTU 29	2.69	Control	0.037	*Ruminococcus_1*
OTU 34	3.60	5th cutting chloroform extract	0.016	*Muribaculaceae_ge*
OTU 37	3.37	Control	0.006	*Lachnospiraceae_NK4A136_group*
OTU 42	3.09	5th cutting chloroform extract	0.006	*Lachnospiraceae_NK4A136_group*
OTU 44	2.50	Control	0.006	*Lachnospiraceae GCA-900066575*
OTU 46	2.92	5th cutting chloroform extract	0.006	*Lachnospiraceae uncultured*
OTU 49	2.25	Control	0.007	*Romboutsia*
OTU 64	2.93	Control	0.004	*Lachnoclostridium*
OTU 66	2.92	Control	0.006	*Lachnospiraceae ASF356*
OTU 74	3.70	Control	0.046	*Faecalibaculum*
OTU 80	3.04	5th cutting chloroform extract	0.025	*Muribaculaceae_ge*
OTU 88	2.68	Control	0.014	*Lachnospiraceae_NK4A136_group*
OTU 95	2.70	5th cutting chloroform extract	0.006	*Ruminococcaceae_UCG-013*
OTU 100	2.67	Control	0.037	*Lachnospiraceae_unclassified*
