# Supplementary material for: Host immunity and the colon microbiota of mice infected with Citrobacter rodentium are beneficially modulated by lipid-soluble extract from late-cutting alfalfa in the early stages of infection
Source: PLoS One. 2020 Jul 16;15(7):e0236106. doi: 10.1371/journal.pone.0236106 (PMC7365448; doi:10.1371/journal.pone.0236106)
Supplement: S3 Table — (PDF) [file pone.0236106.s004.pdf]

S3 Table. Significantly different OTUs in the colon microbiota of healthy mice fed 1st cutting chloroform extract vs. 5th cutting chloroform extract at d14.

OTU	LDA effect size score	Treatment in which OTU is more abundant	p-value	Taxonomy
OTU 1	4.65	5th cutting chloroform extract	0.006	*Muribaculaceae_ge*
OTU 4	4.74	1st cutting chloroform extract	0.037	*Muribaculaceae_ge*
OTU 10	4.74	1st cutting chloroform extract	0.021	*Muribaculaceae_ge*
OTU 12	4.22	1st cutting chloroform extract	0.020	*Muribaculaceae_ge*
OTU 22	3.27	5th cutting chloroform extract	0.022	*Ruminococcaceae_UCG-014*
OTU 24	3.25	1st cutting chloroform extract	0.016	*Lachnospiraceae_NK4A136_group*
OTU 26	3.64	5th cutting chloroform extract	0.010	*Muribaculaceae_ge*
OTU 31	2.42	1st cutting chloroform extract	0.006	*Lachnospiraceae_unclassified*
OTU 34	3.64	5th cutting chloroform extract	0.006	*Muribaculaceae_ge*
OTU 46	2.75	5th cutting chloroform extract	0.037	*Lachnospiraceae*
OTU 56	3.14	1st cutting chloroform extract	0.004	*Lachnospiraceae_unclassified*
OTU 60	2.63	1st cutting chloroform extract	0.037	*Lachnospiraceae_unclassified*
OTU 64	2.50	1st cutting chloroform extract	0.010	*Lachnoclostridium*
OTU 70	3.02	5th cutting chloroform extract	0.010	*Muribaculaceae_ge*
OTU 72	3.20	1st cutting chloroform extract	0.002	*Muribaculaceae_ge*

OTU 76	3.21	1st cutting chloroform extract	0.007	*Muribaculaceae_ge*
OTU 78	3.19	1st cutting chloroform extract	0.002	*Muribaculaceae_ge*
OTU 80	3.12	5th cutting chloroform extract	0.006	*Muribaculaceae_ge*
OTU 83	3.44	1st cutting chloroform extract	0.022	*Muribaculaceae_ge*
OTU 87	3.34	1st cutting chloroform extract	0.022	*Muribaculaceae_ge*
