# Supplementary material for: Host immunity and the colon microbiota of mice infected with Citrobacter rodentium are beneficially modulated by lipid-soluble extract from late-cutting alfalfa in the early stages of infection
Source: PLoS One. 2020 Jul 16;15(7):e0236106. doi: 10.1371/journal.pone.0236106 (PMC7365448; doi:10.1371/journal.pone.0236106)
Supplement: S4 Table — (PDF) [file pone.0236106.s005.pdf]

S4 Table. Significantly different OTUs in the colon microbiota of healthy mice fed the control diet vs. 1st cutting chloroform extract at 4dpi.

OTU	LDA effect size score	Treatment in which OTU is more abundant	p-value	Taxonomy
OTU 3	4.36	Control	0.034	*Bacteroides*
OTU 6	4.28	1st cutting chloroform extract	0.034	*Lactobacillus*
OTU 15	3.81	1st cutting chloroform extract	0.034	*Lachnospiraceae_NK4A136_group*
OTU 25	3.95	1st cutting chloroform extract	0.034	*Lachnospiraceae_UCG-001*
OTU 27	3.28	Control	0.034	*Bacteroides*
OTU 32	4.05	1st cutting chloroform extract	0.028	*Lachnospiraceae_unclassified*
OTU 33	3.01	1st cutting chloroform extract	0.019	*Muribaculaceae_ge*
OTU 43	2.43	1st cutting chloroform extract	0.034	*Lachnospiraceae_unclassified*
OTU 44	2.33	Control	0.034	*GCA-900066575*
OTU 56	3.56	1st cutting chloroform extract	0.034	*Lachnospiraceae_unclassified*
OTU 58	3.40	Control	0.034	*Roseburia*
OTU 65	3.38	1st cutting chloroform extract	0.034	*Lachnospiraceae_UCG-001*
OTU 66	2.48	1st cutting chloroform extract	0.034	*Lachnospiraceae ASF356*
