# Supplementary material for: Host immunity and the colon microbiota of mice infected with Citrobacter rodentium are beneficially modulated by lipid-soluble extract from late-cutting alfalfa in the early stages of infection
Source: PLoS One. 2020 Jul 16;15(7):e0236106. doi: 10.1371/journal.pone.0236106 (PMC7365448; doi:10.1371/journal.pone.0236106)
Supplement: S5 Table — (PDF) [file pone.0236106.s006.pdf]

S5 Table. Significantly different OTUs in the colon microbiota of healthy mice fed the control diet vs. 5th cutting chloroform extract at 4dpi.

OTU	LDA effect size score	Treatment in which OTU is more abundant	p-value	Taxonomy
OTU 1	4.56	Control	0.021	*Muribaculaceae_ge*
OTU 2	4.76	Control	0.021	*Muribaculaceae_ge*
OTU 3	4.44	Control	0.021	*Bacteroides*
OTU 4	5.08	5th cutting chloroform extract	0.021	*Muribaculaceae_ge*
OTU 12	3.98	5th cutting chloroform extract	0.018	*Muribaculaceae_ge*
OTU 15	4.03	5th cutting chloroform extract	0.043	*Lachnospiraceae_NK4A136_group*
OTU 17	3.49	Control	0.021	*Muribaculaceae_ge*
OTU 19	3.51	Control	0.021	*Bifidobacterium*
OTU 21	2.92	Control	0.021	*Parasutterella*
OTU 26	3.48	Control	0.021	*Muribaculaceae_ge*
OTU 27	3.20	Control	0.021	*Bacteroides*
OTU 31	2.66	5th cutting chloroform extract	0.020	*Lachnospiraceae_unclassified*
OTU 33	3.68	5th cutting chloroform extract	0.014	*Muribaculaceae_ge*
OTU 34	3.35	Control	0.021	*Muribaculaceae_ge*
OTU 41	3.58	5th cutting chloroform extract	0.020	*Muribaculaceae_ge*
OTU 45	3.07	5th cutting chloroform extract	0.014	*Muribaculaceae_ge*
OTU 47	3.66	Control	0.021	*Enterobacteriaceae_unclassified*
OTU 56	3.92	5th cutting chloroform extract	0.021	*Lachnospiraceae_unclassified*
OTU 57	2.90	5th cutting chloroform extract	0.014	*Muribaculaceae_ge*
OTU 62	2.83	5th cutting chloroform extract	0.014	*Muribaculaceae_ge*
OTU 70	2.86	Control	0.021	*Muribaculaceae_ge*
OTU 72	2.95	5th cutting chloroform extract	0.014	*Muribaculaceae_ge*

OTU 76	2.65	5th cutting chloroform extract	0.014	*Muribaculaceae_ge*
OTU 77	2.97	5th cutting chloroform extract	0.018	*Muribaculaceae_ge*
OTU 78	2.67	5th cutting chloroform extract	0.014	*Muribaculaceae_ge*
OTU 79	3.34	5th cutting chloroform extract	0.014	*Muribaculaceae_ge*
OTU 80	2.86	Control	0.021	*Muribaculaceae_ge*
OTU 85	3.25	5th cutting chloroform extract	0.014	*Muribaculaceae_ge*
OTU 99	2.72	Control	0.021	*Lachnospiraceae_unclassified*
