# Supplementary material for: Host immunity and the colon microbiota of mice infected with Citrobacter rodentium are beneficially modulated by lipid-soluble extract from late-cutting alfalfa in the early stages of infection
Source: PLoS One. 2020 Jul 16;15(7):e0236106. doi: 10.1371/journal.pone.0236106 (PMC7365448; doi:10.1371/journal.pone.0236106)
Supplement: S6 Table — (PDF) [file pone.0236106.s007.pdf]

S6 Table. Significantly different OTUs in the colon microbiota of healthy mice fed 1st cutting chloroform extract vs. 5th cutting chloroform extract at 4dpi.

OTU	LDA effect size score	Treatment in which OTU is more abundant	p-value	Taxonomy
OTU 4	4.98	5th cutting chloroform extract	0.034	*Muribaculaceae_ge*
OTU 12	4.06	5th cutting chloroform extract	0.034	*Muribaculaceae_ge*
OTU 30	2.39	1st cutting chloroform extract	0.034	*Lachnoclostridium*
OTU 32	4.04	1st cutting chloroform extract	0.034	*Lachnospiraceae_unclassified*
OTU 33	3.71	5th cutting chloroform extract	0.034	*Muribaculaceae_ge*
OTU 41	3.59	5th cutting chloroform extract	0.034	*Muribaculaceae_ge*
OTU 58	2.48	5th cutting chloroform extract	0.034	*Roseburia*
OTU 65	3.32	1st cutting chloroform extract	0.034	*Lachnospiraceae_UCG-001*
OTU 72	3.12	5th cutting chloroform extract	0.034	*Muribaculaceae_ge*
OTU 77	2.85	5th cutting chloroform extract	0.034	*Muribaculaceae_ge*
OTU 78	2.81	5th cutting chloroform extract	0.032	*Muribaculaceae_ge*
OTU 79	3.46	5th cutting chloroform extract	0.034	*Muribaculaceae_ge*
OTU 82	2.58	1st cutting chloroform extract	0.034	*Enterorhabdus*
OTU 85	3.37	5th cutting chloroform extract	0.034	*Muribaculaceae_ge*
OTU 89	2.56	1st cutting chloroform extract	0.034	*Lachnospiraceae_UCG-006*
OTU 93	2.72	5th cutting chloroform extract	0.032	*Ruminococcaceae_UCG-014*
