# Supplementary material for: Host immunity and the colon microbiota of mice infected with Citrobacter rodentium are beneficially modulated by lipid-soluble extract from late-cutting alfalfa in the early stages of infection
Source: PLoS One. 2020 Jul 16;15(7):e0236106. doi: 10.1371/journal.pone.0236106 (PMC7365448; doi:10.1371/journal.pone.0236106)
Supplement: S7 Table — (PDF) [file pone.0236106.s008.pdf]

S7 Table. Significantly different OTUs in the colon microbiota of healthy mice fed the control diet vs. 1st cutting chloroform extract at 14dpi.

OTU	LDA effect size score	Treatment in which OTU is more abundant	p-value	Taxonomy
OTU 7	4.49	Control	0.021	*Lachnospiraceae_NK4A136_group*
OTU 12	2.87	Control	0.018	*Muribaculaceae_ge*
OTU 13	3.32	1st cutting chloroform extract	0.021	*Turicibacter*
OTU 15	4.04	1st cutting chloroform extract	0.021	*Lachnospiraceae_NK4A136_group*
OTU 19	3.82	1st cutting chloroform extract	0.021	*Bifidobacterium*
OTU 31	3.71	Control	0.020	*Lachnospiraceae_unclassified*
OTU 49	3.92	1st cutting chloroform extract	0.043	*Romboutsia*
OTU 50	3.01	Control	0.043	*Lachnoclostridium*
OTU 65	2.46	Control	0.038	*Lachnospiraceae_UCG-001*
OTU 69	2.10	Control	0.043	*Lachnospiraceae uncultured*
OTU 97	2.73	Control	0.018	*Clostridiales_vadinBB60_group_ge*
