# Supplementary material for: Host immunity and the colon microbiota of mice infected with Citrobacter rodentium are beneficially modulated by lipid-soluble extract from late-cutting alfalfa in the early stages of infection
Source: PLoS One. 2020 Jul 16;15(7):e0236106. doi: 10.1371/journal.pone.0236106 (PMC7365448; doi:10.1371/journal.pone.0236106)
Supplement: S8 Table — (PDF) [file pone.0236106.s009.pdf]

S8 Table. Significantly different OTUs in the colon microbiota of healthy mice fed the control diet vs. 5th cutting chloroform extract at 14dpi.

OTU	LDA effect size score	Treatment in which OTU is more abundant	p-value	Taxonomy
OTU 4	3.27	5th cutting chloroform extract	0.021	*Muribaculaceae_ge*
OTU 13	3.82	5th cutting chloroform extract	0.021	*Turicibacter*
OTU 28	3.79	5th cutting chloroform extract	0.043	*Lachnospiraceae_unclassified*
OTU 35	4.10	5th cutting chloroform extract	0.021	*Lachnospiraceae_NK4A136_group*
OTU 42	3.19	5th cutting chloroform extract	0.021	*Lachnospiraceae_NK4A136_group*
OTU 45	2.37	5th cutting chloroform extract	0.020	*Muribaculaceae_ge*
OTU 57	2.35	5th cutting chloroform extract	0.014	*Muribaculaceae_ge*
OTU 58	2.94	Control	0.014	*Roseburia*
OTU 59	2.81	Control	0.021	*Lachnospiraceae_NK4A136_group*
OTU 62	2.06	5th cutting chloroform extract	0.047	*Muribaculaceae_ge*
OTU 92	2.57	5th cutting chloroform extract	0.021	*Clostridium_sensu_stricto_1*
OTU 97	2.56	Control	0.042	*Clostridiales_vadinBB60_group_ge*
