# Supplementary material for: Host immunity and the colon microbiota of mice infected with Citrobacter rodentium are beneficially modulated by lipid-soluble extract from late-cutting alfalfa in the early stages of infection
Source: PLoS One. 2020 Jul 16;15(7):e0236106. doi: 10.1371/journal.pone.0236106 (PMC7365448; doi:10.1371/journal.pone.0236106)
Supplement: S10 Table — (PDF) [file pone.0236106.s011.pdf]

S10 Table. Significantly different OTUs in the colon microbiota of healthy mice fed the control diet vs. 1st cutting chloroform extract at 21dpi.

OTU	LDA effect size score	Treatment in which OTU is more abundant	p-value	Taxonomy
OTU 10	3.60	Control	0.021	*Muribaculaceae_ge*
OTU 15	3.67	Control	0.018	*Lachnospiraceae_NK4A136_group*
OTU 17	3.10	Control	0.043	*Muribaculaceae_ge*
OTU 18	3.46	Control	0.021	*Anaeroplasma*
OTU 25	3.58	Control	0.043	*Lachnospiraceae_UCG-001*
OTU 32	3.38	Control	0.018	*Lachnospiraceae_unclassified*
OTU 42	3.33	Control	0.021	*Lachnospiraceae_NK4A136_group*
OTU 43	3.27	Control	0.038	*Lachnospiraceae_unclassified*
OTU 48	2.79	Control	0.020	*Lachnoclostridium*
OTU 58	3.21	1st cutting chloroform extract	0.042	*Roseburia*
OTU 65	3.09	Control	0.020	*Lachnospiraceae_UCG-001*
OTU 67	2.16	1st cutting chloroform extract	0.047	*Mollicutes_RF39_ge*
OTU 83	2.33	Control	0.014	*Muribaculaceae_ge*
OTU 84	2.49	Control	0.018	*Lachnospiraceae A2*
OTU 87	2.26	Control	0.014	*Muribaculaceae_ge*
