# Supplementary material for: Host immunity and the colon microbiota of mice infected with Citrobacter rodentium are beneficially modulated by lipid-soluble extract from late-cutting alfalfa in the early stages of infection
Source: PLoS One. 2020 Jul 16;15(7):e0236106. doi: 10.1371/journal.pone.0236106 (PMC7365448; doi:10.1371/journal.pone.0236106)
Supplement: S11 Table — (PDF) [file pone.0236106.s012.pdf]

S11 Table. Significantly different OTUs in the colon microbiota of healthy mice fed the control diet vs. 5th cutting chloroform extract at 21dpi.

OTU	LDA effect size score	Treatment in which OTU is more abundant	p-value	Taxonomy
OTU 3	4.26	5th cutting chloroform extract	0.043	*Bacteroides*
OTU 8	4.45	5th cutting chloroform extract	0.021	*Akkermansia*
OTU 10	3.51	Control	0.020	*Muribaculaceae_ge*
OTU 15	3.85	Control	0.018	*Lachnospiraceae_NK4A136_group*
OTU 18	3.37	Control	0.042	*Anaeroplasma*
OTU 27	3.03	5th cutting chloroform extract	0.043	*Bacteroides*
OTU 32	3.47	Control	0.018	*Lachnospiraceae_unclassified*
OTU 35	3.41	Control	0.042	*Lachnospiraceae_NK4A136_group*
OTU 36	2.80	Control	0.021	*Oscillibacter*
OTU 38	2.87	Control	0.021	*Lachnospiraceae_NK4A136_group*
OTU 40	3.03	Control	0.020	*Ruminiclostridium_5*
OTU 42	3.40	Control	0.021	*Lachnospiraceae_NK4A136_group*
OTU 48	3.12	Control	0.018	*Lachnoclostridium*
OTU 53	2.96	Control	0.043	*Ruminococcaceae_unclassified*
OTU 55	2.88	Control	0.042	*Ruminiclostridium*
OTU 59	2.88	Control	0.021	*Lachnospiraceae_NK4A136_group*
OTU 63	2.78	Control	0.021	*Lachnospiraceae_unclassified*
OTU 64	2.98	Control	0.042	*Lachnoclostridium*
OTU 65	3.62	Control	0.038	*Lachnospiraceae_UCG-001*
OTU 71	3.16	Control	0.047	*Lachnospiraceae_unclassified*
OTU 73	2.64	Control	0.021	*Lachnospiraceae uncultured*
OTU 76	2.71	Control	0.043	*Muribaculaceae_ge*
OTU 78	2.65	Control	0.043	*Muribaculaceae_ge*
OTU 83	2.18	Control	0.014	*Muribaculaceae_ge*
OTU 84	2.40	Control	0.038	*Lachnospiraceae A2*
OTU 86	2.49	Control	0.020	*Lachnoclostridium*
OTU 87	2.13	Control	0.014	*Muribaculaceae_ge*
OTU 89	2.25	Control	0.021	*Lachnospiraceae_UCG-006*
OTU 98	2.61	Control	0.021	*Lachnospiraceae_unclassified*
