# Supplementary material for: Host immunity and the colon microbiota of mice infected with Citrobacter rodentium are beneficially modulated by lipid-soluble extract from late-cutting alfalfa in the early stages of infection
Source: PLoS One. 2020 Jul 16;15(7):e0236106. doi: 10.1371/journal.pone.0236106 (PMC7365448; doi:10.1371/journal.pone.0236106)
Supplement: S12 Table — (PDF) [file pone.0236106.s013.pdf]

S12 Table. Significantly different OTUs in the colon microbiota of healthy mice fed 1st cutting chloroform extract vs. 5th cutting chloroform extract at 21dpi.

OTU	LDA effect size score	Treatment in which OTU is more abundant	p-value	Taxonomy
OTU 28	3.28	1st cutting chloroform extract	0.021	*Lachnospiraceae_unclassified*
OTU 36	2.97	1st cutting chloroform extract	0.021	*Oscillibacter*
OTU 40	2.75	1st cutting chloroform extract	0.020	*Ruminiclostridium_5*
OTU 53	2.50	1st cutting chloroform extract	0.021	*Ruminococcaceae_unclassified*
OTU 58	3.14	1st cutting chloroform extract	0.043	*Roseburia*
OTU 59	2.66	1st cutting chloroform extract	0.043	*Lachnospiraceae_NK4A136_group*
OTU 81	2.59	1st cutting chloroform extract	0.042	*Clostridiales_vadinBB60_group_ge*
OTU 86	2.26	1st cutting chloroform extract	0.020	*Lachnoclostridium*
OTU 89	2.18	1st cutting chloroform extract	0.021	*Lachnospiraceae_UCG-006*
OTU 98	2.62	1st cutting chloroform extract	0.021	*Lachnospiraceae_unclassified*
OTU 99	2.36	1st cutting chloroform extract	0.047	*Lachnospiraceae_unclassified*
